# Multiparametric magnetic resonance imaging features of a canine glioblastoma model

**DOI:** 10.1371/journal.pone.0254448

**Published:** 2021-07-09

**Authors:** Seunghyun Lee, Seung Hong Choi, Hye Rim Cho, Jaemoon Koh, Chul-Kee Park, Tomotsugu Ichikawa

**Affiliations:** 1 Department of Radiology, Seoul National University Hospital, Seoul, Republic of Korea; 2 Department of Radiology, Seoul National University College of Medicine, Seoul, Republic of Korea; 3 Center for Nanoparticle Research, Institute for Basic Science, Seoul, Republic of Korea; 4 School of Chemical and Biological Engineering, Seoul National University, Seoul, Republic of Korea; 5 Department of Pathology, Seoul National University Hospital, Seoul, Republic of Korea; 6 Department of Neurosurgery, Seoul National University College of Medicine, Seoul, Republic of Korea; 7 Department of Neurological Surgery, Okayama University Graduate School of Medicine, Dentistry, and Pharmaceutical Sciences, Okayama, Japan; Henry Ford Health System, UNITED STATES

## Abstract

**Purpose:**

To assess glioblastoma multiforme (GBM) formation with similar imaging characteristics to human GBM using multiparametric magnetic resonance imaging (MRI) in an orthotopic xenograft canine GBM model.

**Materials and methods:**

The canine GBM cell line J3T1 was subcutaneously injected into 6-week-old female BALB/c nude mice to obtain tumour fragments. Tumour fragments were implanted into adult male mongrel dog brains through surgery. Multiparametric MRI was performed with conventional MRI, diffusion-weighted imaging, and dynamic susceptibility contrast-enhanced perfusion-weighted imaging at one week and two weeks after surgery in a total of 15 surgical success cases. The presence of tumour cells, the necrotic area fraction, and the microvessel density (MVD) of the tumour on the histologic specimen were assessed. Tumour volume, diffusion, and perfusion parameters were compared at each time point using Wilcoxon signed-rank tests, and the differences between tumour and normal parenchyma were compared using unpaired t-tests. Spearman correlation analysis was performed between the imaging and histologic parameters.

**Results:**

All animals showed a peripheral enhancing lesion on MRI and confirmed the presence of a tumour through histologic analysis (92.3%). The normalized perfusion values did not show significant decreases through at least 2 weeks after the surgery (*P* > 0.05). There was greater cerebral blood volume and flow in the GBM than in the normal-appearing white matter (1.46 ± 0.25 vs. 1.13 ± 0.16 and 1.30 ± 0.22 vs. 1.02 ± 0.14; *P* < 0.001 and *P* < 0.001, respectively). The MVD in the histologic specimens was correlated with the cerebral blood volume in the GBM tissue (r = 0.850, *P* = 0.004).

**Conclusion:**

Our results suggest that the canine GBM model showed perfusion imaging characteristics similar to those of humans, and it might have potential as a model to assess novel technical developments for GBM treatment.

## Introduction

Glioblastoma multiforme (GBM) is the most common malignant primary brain tumour and carries a poor prognosis [1]. The poor prognosis may be attributed to the infiltrative nature of GBM itself, the limitations of therapy, and an incomplete understanding of the pathophysiology of the tumour [2]. Therefore, it is essential to obtain evidence for applying new diagnostic and treatment methods in preclinical GBM models similar to human GBM.

However, it is challenging to apply the knowledge obtained from the rodent models widely used in preclinical studies to human GBM. The small animal GBM models’ limitation is that they are too small to evaluate newly applied diagnostic or treatment devices [3–6]. The canine GBM model, one of the large animals, is a possible preclinical model; it is an ideal model that more closely resembles human GBM than the small animal models in terms of brain size [3–6]. The preclinical testing of novel devices for effective treatment requires easier access to diagnostic and therapeutic manipulations in animal models similar in size to humans. Likewise, a multiparametric magnetic resonance imaging (MRI) approach for physiologic imaging and treatment response assessment in GBM can now be used for both primary brain tumours in dogs and humans, which could further facilitate the application of new methods in human patients.

The orthotopic xenograft canine model may yield physiologic information about the tumour itself through the multiparametric MRI used in current clinical imaging due to having sufficient brain size for image acquisition [6, 7]. The MRI features of human GBMs show peripheral enhancing necrotic masses and increased tumour perfusion [8–12]. In particular, dynamic susceptibility contrast-enhanced perfusion-weighted imaging (DSC-PWI) can provide *in vivo* physiological status, such as the abnormal blood vessel formation with higher cerebral blood volume (CBV) observed in human GBM [13–15].

Therefore, this study aimed to assess GBM formation with similar imaging characteristics to human GBM using multiparametric MRI and pathological results in an orthotopic xenograft canine GBM model.

## Materials and methods

This study was approved by the Institutional Animal Care and Use Committee of the Seoul National University Hospital (IACUC; No. 14-0156-C2A3) and was performed in accordance with the Guide from our IACUC and the National Institute of Health Guide for the Care and Use of Laboratory Animals. All animals were maintained in an AAALAC International (#001169) accredited facility in accordance with Guide for the Care and Use of Laboratory Animals 8th edition, NRC (2010).

### Cell culture

The canine glioblastoma cell line J3T1, derived from the canine glioma cell line J3T, was provided by the Okayama University School of Medicine, School of Dentistry, after obtaining research ethics approval. The cell lines were maintained in Roswell Park Memorial Institute (RPMI) medium with 10% fetal bovine serum (FBS) and a 1% penicillin/streptomycin mixture (Gibco, Grand Island, NY, USA) at 37°C. Cell viability was assessed with trypan blue staining to confirm a cell viability of > 90% before tumour implantation.

### Preparation of tumour fragments for canine tumour implantation

**Fig 1:**
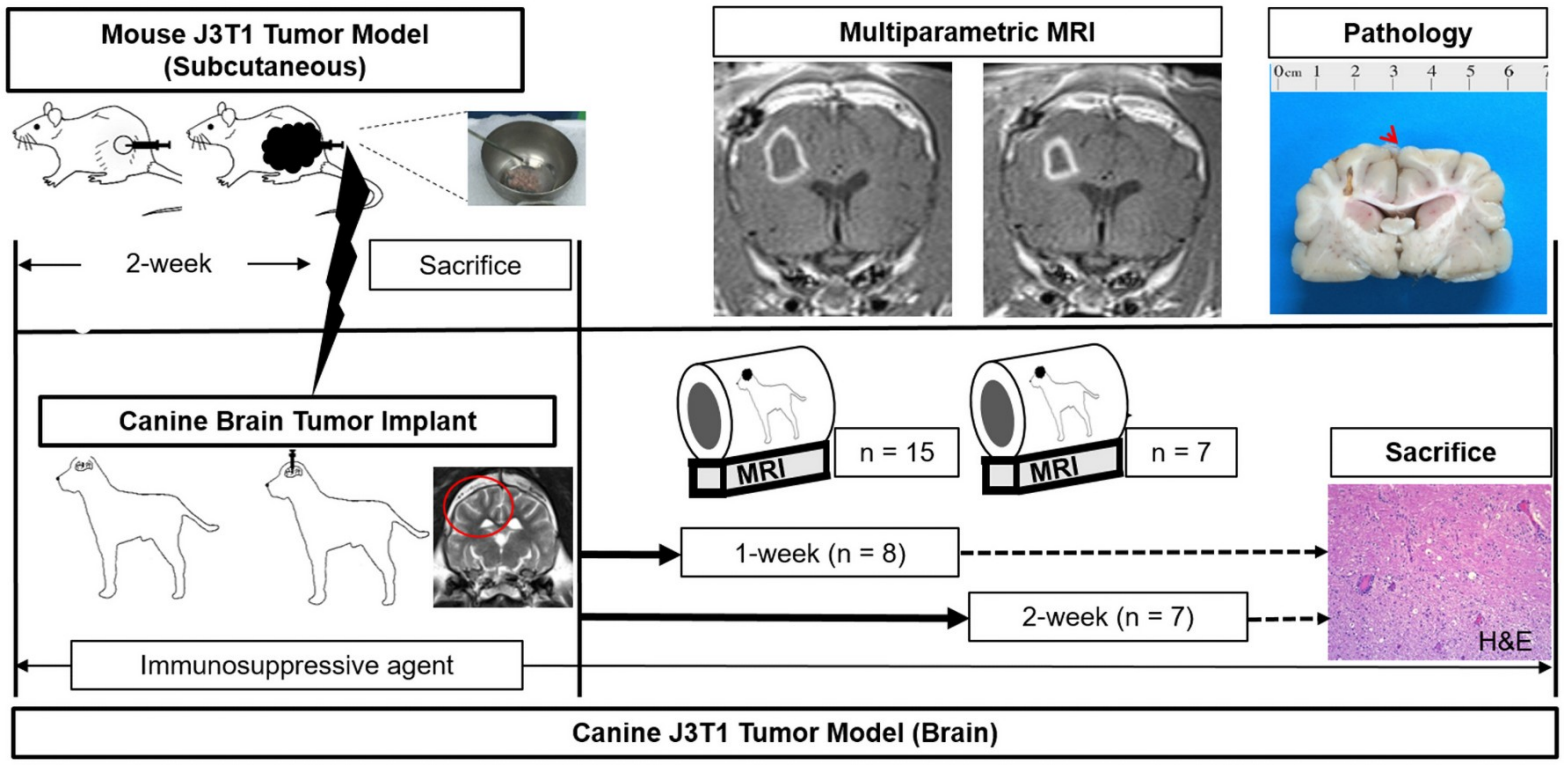
Experimental design. J3T1 cells were subcutaneously injected into the flank area of BALB/c nude mice. After two weeks, the mice were sacrificed to obtain viable tumour fragments. Preimplantation, dogs were treated with immunosuppressive agents for two weeks, and tumour fragments from the mice were injected into the right frontoparietal region of the brain parenchyma. After one week, postimplantation MRI with diffusion-weighted and perfusion-weighted imaging was performed every week until sacrifice. Immunosuppressive agents were also administered to the implanted dogs until sacrifice. Fifteen canine models were successfully created, and brain images from eight and seven subjects were obtained at 1 week and 2 weeks, respectively. After the follow-up imaging study, the brains were extracted for histological analysis.

The experimental design is summarized in Fig 1. J3T1 cells were prepared in 100 mL serum-free RPMI medium. Under intraperitoneal general anaesthesia using a mixture of 5 mg/kg zolazepam (Zoletil; Virbac, Carros, France) and xylazine hydrochloride (Rompun 2%; Bayer Korea, Seoul, Korea), cells (2 × 10^5^/100 μL medium/mouse) were subcutaneously injected into the flanks of 6-week-old female BALB/c nude mice (Koatech, Korea). After 2 weeks, the mice were sacrificed with a lethal dose of sodium pentobarbital (100 mg/kg body weight, intraperitoneal) to obtain tumour fragments. The tumour fragments were excised to a diameter of 3–5 mm, washed with phosphate-buffered saline (PBS), and suspended in 50% basement membrane matrix (Matrigel, BD Bioscience, USA) in an icebox.

### Immune system suppression for canine tumour implantation

Eighteen healthy male mongrel dogs (20 weeks old; range 10.0–12.0 kg) were used in this study and were purchased from the International Laboratory Animal Center (Pocheon, Gyeonggi-do, Korea). They were housed separately in stainless steel cages (W 895 × L 795 × H 765 mm) in an environmentally controlled room (temperature 23 ± 3°C, relative humidity 55 ± 15%, ventilation frequency 10–20 times/hr, light cycle 8 am to 8 pm, illumination 150 to 300 lux). Food and sterilized water were available ad libitum.

Three immunosuppressive agents were used to suppress the immune system in the experimental dogs before tumour implantation. A regimen based on one previously used canine gliosarcoma and cavernous sinus tumour model was modified and optimized through preliminary experiments [16, 17]. The experimental dogs were treated with an oil-based formulation of cyclosporine (Sandimmune; Novartis Pharm., UK) (20 mg/kg orally twice daily). In addition, 50 mg of azathioprine (Azaprine Tab., Korea United Pharm. Inc., Seoul, Korea) and 10 mg of prednisolone (Solondo Tab., YuhanMedica, Seoul, Korea) were mixed with normal saline and administered orally twice daily. The immunosuppressive agents were continuously administered from two weeks before tumour transplantation until the end of the study. We monitored the dogs daily to check for complications from these persistent immunosuppressive agents.

### Canine tumour implantation procedure

The surgical procedure for canine tumour implantation was performed under general anaesthesia. The animals were sedated with intravenous administration of a 1:1 combination of tiletamine hydrochloride and zolazepam (Zoletil; Virbac, Carros, France) and xylazine hydrochloride (Rompun 2%; Bayer Korea, Seoul, Korea) at a concentration of 5 mg/kg. Then, respiratory anaesthesia was maintained with a ventilator using 0.5 to 3% isoflurane during the surgical procedure.

All procedures were performed under aseptic techniques after disinfecting the right frontoparietal region and covering it with a sterilized cloth. A craniotomy was performed to create a small burr hole (0.3–0.5 cm in diameter) using a handpiece drill (STRONG 204; Saeshin Precision Co., Ltd., Korea). CSF pulsation in the subdural space was confirmed through the hole, and the prepared tumour fragment was implanted using an 18-gauge spinal needle. Procedural bleeding was prevented and controlled using bone wax (ETHW31G, Ethicon, USA) and N-butyl cyanoacrylate (Histoacryl, Braun, Germany). The overlaying muscle and skin were then closed using a 3–0 Vicryl suture and a 4–0 nylon suture. All dogs were given intramuscular injections of cephazolin (cefazolin; Chong Kun Dang Pharm. Co., Korea; 20 mg/kg/day) to prevent infection for 7 days, and the surgical sites were disinfected daily for 7 days after surgery.

### Image acquisition

MRI scans were performed using a 3.0 T MR imaging system (Magnetom Trio; Siemens Medical Solutions, Germany) with a human head coil. All MRI examinations were performed with the dogs in the supine position and included the entire brain. The animals were sedated with the same intravenous sedative drug administration method as used for the surgery during all MRI examinations.

The MRI sequences included coronal turbo-spin echo T2-weighted imaging (T2WI), coronal gradient-echo T1-weighted imaging (T1WI), and coronal contrast-enhanced T1WI (CE-T1WI). CE T1WI was performed after an intravenous injection of 0.2 mL/kg gadoteric acid (Dotarem; Guerbet, France) via the cephalic vein. MRI parameters were as follows: 5160/91 ms/131°/640 × 290/5 mm (TR/TE/FA/matrix/section thickness) for T2WI and 990/9.8 ms/70°/384 × 222/1.5 mm for T1WI and CE-T1WI.

Diffusion-weighted imaging (DWI) was obtained with a single-shot spin-echo echo-planar imaging sequence in the coronal plane before contrast injection, similar to the imaging method in humans [15, 18]. DWI images were acquired under the following conditions: a TR/TE of 12000/80 ms at b = 0 and 1000 sec/mm^2^, 38 sections, a 3-mm section thickness, a 0.9-mm intersection gap, an FOV of 280 × 280 mm, a matrix of 192 × 192, and three signal averages [15]. An average apparent diffusion coefficient (ADC) map made from three orthogonal data on the DWI image was created using the MRI unit’s built-in software [15].

DSC-PWI was acquired in the same way as that used in humans using a single-shot gradient-echo imaging sequence during the contrast agent’s intravenous injection [14, 15, 18, 19]. DSC-PWI images were acquired under the following conditions: a TR/TE of 1500/30 ms, 20 sections, a 5 mm-section thickness, a 1-mm intersection gap, an FOV of 240 × 240 mm, a matrix of 128 × 128, and an FA of 90°. Each section consisted of 60 images with equal intervals and image parameters, resulting in 1200 images. After obtaining four to five sections of images, we injected the MRI contrast agent gadobutrol using an MR-compatible power injector (Spectris; Medrad, Pittsburgh, PA, USA) under the following conditions: 0.1 mmol/kg body weight and 2 mL/sec rate [15]. After contrast injection, a 15 mL bolus of saline was administered at the same injection rate.

Image acquisition was performed at baseline before tumour implantation and every week after surgery until sacrifice. The dogs were assigned to one of the two groups with one-week and two-week follow-ups because of gingival hypertrophy due to long-term use of cyclosporine for more than two weeks in our preliminary experiment.

### Image analysis

**Fig 2:**
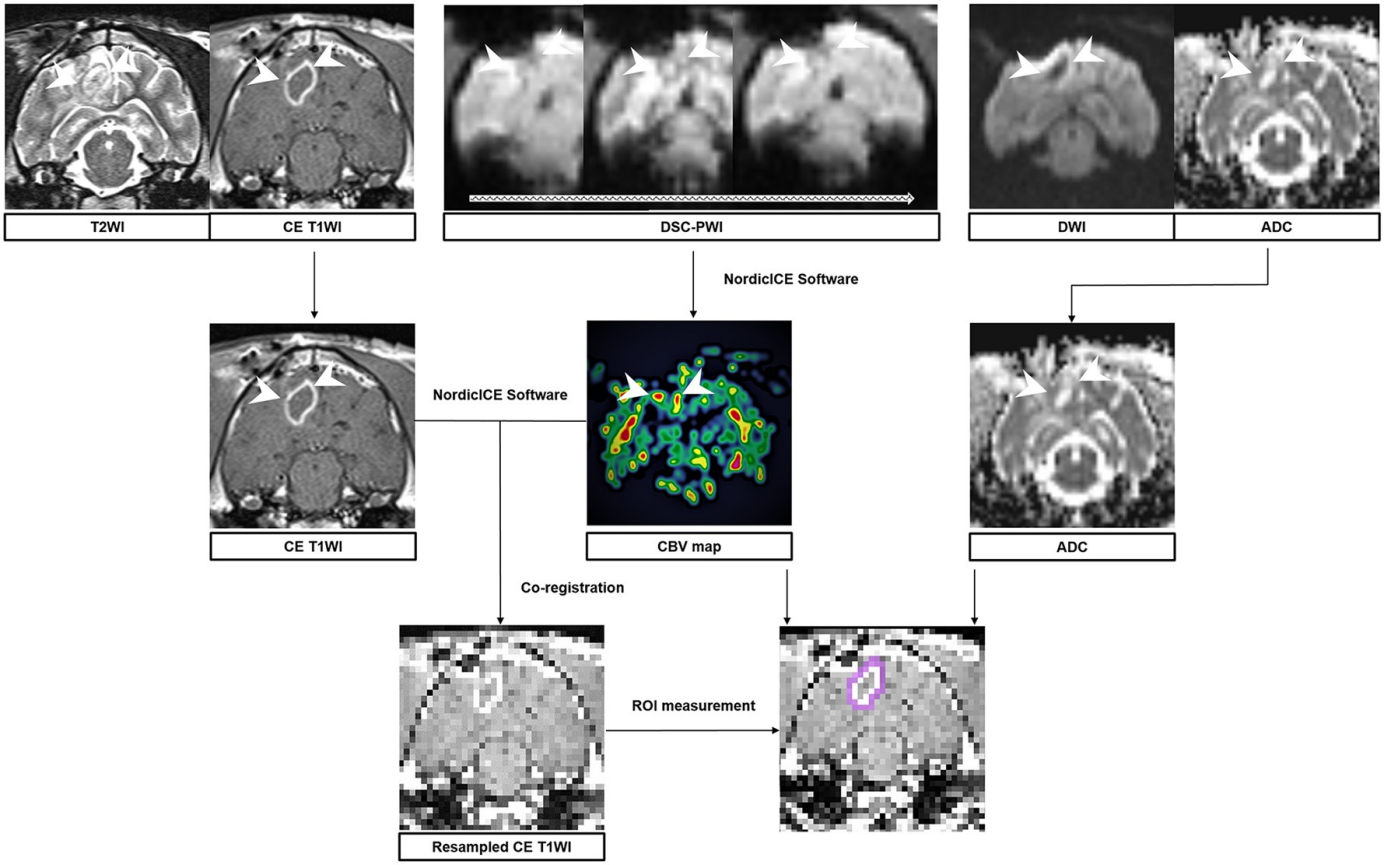
Flow chart of the quantitative image analysis. Flow chart of the quantitative image analysis. A region of interest (ROI) was manually selected in each section of the enhancing lesions and semiautomatically coregistered with the relative cerebral blood volume map (rCBV) and coronal contrast-enhanced T1-weighted images. The volume of interest is determined by the summation of the ROIs from each slice; the rCBV values and apparent diffusion coefficient (ADC) values for the entire enhancing lesion are obtained. The relative perfusion values and ADC values in the normal-appearing white matter and cerebellum are also derived. The normalized perfusion values and ADC values were calculated by dividing each tumour and normal-appearing white matter by the corresponding cerebellar values.

The image analysis methods are presented in Fig 2. Both DSC-PWI and ADC maps were processed using commercialized software (Nordic ICE, NordicNeuroLab), in which CE-T1WI was used for coregistration. The relative CBV (rCBV) maps were generated with established tracer kinetic models applied to the first-pass data [20, 21]. The ΔR2* (1/T2*) curve was mathematically corrected to a gamma-variate function, which approximates the first-pass response that occurs when there is no recycling or leakage to reduce the recirculation effect [22]. The dynamic curves were mathematically corrected to reduce the effect of contrast agent leakage [23]. Coregistration between the rCBV map and CE-T1WI was performed to minimize the differences in slice thickness between images that were automatically corrected by the reslicing and coregistration method, which was based on underlying images and structural images.

One investigator (S.L., 9 years of experience in neuroradiology) drew the regions of interest (ROIs) that contained the entire tumour on every continuous section of the coregistered images. Tumour boundaries were defined with reference to the high-signal intensity areas thought to represent tumour tissue on CE-T1WI. The ROIs included all contrast-enhancing areas, and the values were automatically obtained with the software by summing up all values from each coregistered axial slice, which were then averaged. After obtaining the total voxel values of the rCBV of each tumour, the mean rCBV of each tumour was calculated. In addition, the rCBV values in the normal-appearing white matter contralateral to the enhancing lesion and in the cerebellum were measured for comparison with the rCBV of the tumour. On a pixel-by-pixel basis, the normalized CBV (nCBV) maps were calculated by dividing every relative CBV value by those for unaffected white matter using the NordicICE software package, and all of the pixels from the entire relative CBV map were normalized relative to the mean value of the ROI placed on the contralateral normal-appearing white matter, resulting in the nCBV map. For the comparison of perfusion status between tumours and normal-appearing white matter, the nCBV values, which were calculated on the tumour and normal-appearing white matter by dividing the cerebellum’s value, were obtained and then compared [24]. Other perfusion parameters, such as the relative cerebral blood flow, time-to-peak, and mean transit time, were normalized using the same method described, yielding the normalized cerebral blood flow (nCBF), normalized time-to-peak (nTTP), and normalized mean-transit-time (nMTT). The ADC values in the tumour, normal-appearing white matter, and cerebellum were also measured, and the normalized apparent diffusion coefficient (nADC) values were also calculated using the same method as the nCBV calculation.

### Histopathologic analysis

All of the dogs were sacrificed with an overdose of intravenous pentobarbital sodium (Pentothal; Choong Wae Pharmacy, Seoul, Korea). The specimens were fixed in 10% neutral buffered formalin for one week and then sliced along the coronal plane for a full examination. The specimens were embedded in paraffin and cut into 4-μm sections. Each section was stained with Harris’ haematoxylin solution and eosin Y (H&E) (Sigma, St. Louis, MO, USA) to assess the presence of tumour cells and changes in the vasculature. A pathologist (J.K., 9 years of experience in neuropathology) who was blinded to the experimental information reviewed the specimens. The presence of tumour cells was assessed first, and then the necrotic area fraction (%) from the H&E-stained sections was calculated by dividing the area of the largest cross-section by the area of necrosis in a lower-power field (×40). To obtain the microvessel density (MVD) of the tumour, hot spots, i.e., areas of higher vascular density than the rest of the tissue, were chosen in three random fields per individual tumour section in a low-power field (×40), from which the vessels were counted at high magnification (×400).

### Statistical analysis

All statistical analyses were performed using a commercial software program (MedCalc version 13.1.0.0, MedCalc Software). A *P-*value < 0.05 was considered statistically significant. The Wilcoxon signed-rank test was utilized to evaluate the differences in tumour volume, normalized ADC values, and normalized perfusion values on the 2-week follow-up MRI. Additionally, unpaired Student’s t-tests were performed to compare the ADC and perfusion values between the tumour and normal-appearing white matter. Spearman correlation analysis was performed between the perfusion parameters and MVD.

## Results

### Successful development of the orthotopic xenograft canine GBM model

The results of the development of the orthotopic xenograft canine GBM model are summarized in Table 1. Among the 18 surgical attempts, a total of 15 surgical successes were obtained for the orthotopic xenograft canine GBM model. One case with abscess and two intraventricular extending seeding cases were found on the 1-week follow-up MRI, immediately sacrificed, and excluded from the result data. All 15 subjects showed a peripheral enhancing lesion on the 1-week follow-up MRI. The follow-up period was 1 week for eight subjects and 2 weeks for the other seven subjects (Fig 1). The final success (92.3%) was defined as the presence of a tumour in the implanted lesion on the histologic analysis.

**Table 1:** Canine experimental glioblastoma multiforme model formation.

Parameters	
Number of canine GBM model successes	15
Imaging success at 1-week	15/15 (100.0%)
Presence of tumour on histopathology	12/13 (92.3%)*
Follow-up period	
1-week	8 (53.3%)
2-week	7 (46.7%)

Note—

*Two specimens were unavailable due to tissue injury during the brain extraction procedure and histological process.

### Imaging characteristics of the canine GBM model

The serial follow-up imaging characteristics are summarized in Table 2. Appropriate imaging data were obtained at the 1-week follow-up from 12 subjects. Inadequate imaging data at week one were due to artefacts from the application of bleeding treatment material such as N-butyl cyanoacrylate (n = 2) and damage to the raw image data (n = 1), leading to severe imaging artefacts. The tumour volume slightly decreased from the 1-week to the 2-week follow-up (*P* = 0.043). Neither the nCBV nor nTTP value showed significant serial decreases through the 2-week follow-up imaging (all *P* > 0.05). However, there was a slight decrease in the nCBF and an increase in the nMTT value at the 2-week follow-up (*P* = 0.043 and *P* = 0.043). Additionally, there was no significant difference in the nADC value among the follow-up images (*P* = 0.500).

**Table 2:** Serial follow-up imaging characteristics of the canine GBM model.

Parameters	1-week	2-week	P-value*
Number of imaging scans obtained	15	7	
Adequate images	12	5	
Inadequate images	3	2	
Tumour volume (cm^3^)	1.13 ± 0.68	0.75 ± 0.58	**0.043**
Perfusion parameter			
nCBV	1.52 ± 0.21	1.38 ± 0.34	0.225
nCBF	1.42 ± 0.17	1.14 ± 0.22	**0.043**
nTTP	0.85 ± 0.11	0.84 ± 0.07	0.492
nMTT	0.95 ± 0.16	1.01 ± 0.12	**0.043**
Diffusion parameter			
nADC	0.97 ± 0.14	1.03 ± 0.13	0.500

Note—Except where indicated, the data are the means ± standard deviations.

*P-value was determined using the Wilcoxon signed-rank test between the 1-week and 2-week examinations for the seven subjects with 2-week images. nCBV = normalized cerebral blood volume. nCBF = normalized cerebral blood flow. nTTP = normalized time-to-peak. nMTT = normalized mean-transit-time.

The differences in perfusion and diffusion parameters in the GBM and normal-appearing white matter are summarized in Table 3. In terms of the normalized perfusion parameters, there was an increase in the nCBV and nCBF in the GBM (1.46 ± 0.25 vs. 1.13 ± 0.16 and 1.30 ± 0.22 vs. 1.02 ± 0.14; *P* < 0.001 and *P* < 0.001, respectively). However, a significant difference was not observed between the GBM and normal groups in either the ADC or nADC values.

**Table 3:** Imaging characteristics of the canine GBM model.

Parameters	GBM	NAWM	P-value*
Number of adequate image scans^†^	17		
Perfusion parameter			
nCBV	1.46 ± 0.25	1.13 ± 0.16	**< 0.001**
nCBF	1.30 ± 0.22	1.02 ± 0.14	**< 0.001**
nTTP	0.86 ± 0.08	0.87 ± 0.08	0.636
nMTT	1.01 ± 0.17	1.01 ± 0.03	0.991
Diffusion parameter			
nADC	1.01 ± 0.12	1.06 ± 0.08	0.166

Note—Except where indicated, the data are the means ± standard deviations.

*P-value was determined using the unpaired t-test.

^†^The total number of adequate brain MRI scans was 17 at the 1-week and 2-week follow-ups. GBM = glioblastoma multiforme. NAWM = normal-appearing white matter contralateral to the tumour. nCBV = normalized cerebral blood volume. nCBF = normalized cerebral blood flow. nTTP = normalized time-to-peak. nMTT = normalized mean-transit-time. nADC = normalized apparent diffusion coefficient.

**Fig 3:**
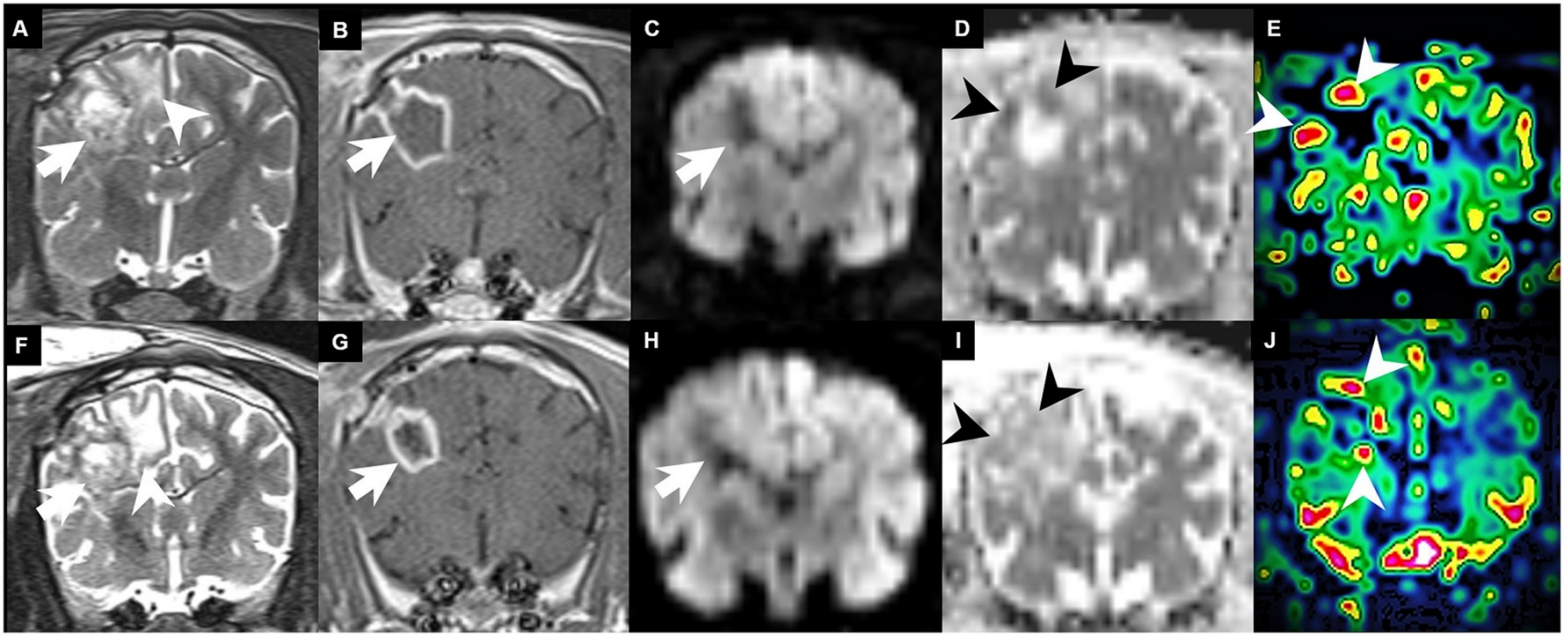
Longitudinal follow-up imaging in a canine brain tumour model. **(A)** After one week of surgery, a tumour was identified at the right frontoparietal area (arrow) with peripheral oedema (arrowhead) on the T2-weighted image. **(B)** The implanted glioblastoma multiforme (GBM) shows a large necrotic mass with peripheral enhancement similar to that seen in humans (arrow) on the contrast-enhanced T1-weighted image. **(C-D)** The mass shows diffusion restriction at the peripheral enhancing portion of the tumour (arrowhead) on the diffusion-weighted image (C) and apparent diffusion coefficient image (D). **(E)** There was also increased CBV (arrowhead) in the peripheral enhancing portion of the GBM on the dynamic susceptibility contrast-enhanced perfusion-weighted image. **(F-G)** On the two-week follow-up MRI, the peripheral enhancing lesion persists (arrow) with perilesional oedema (arrowhead) on the T2-weighted image (F) and contrast-enhanced T1-weighted image (G), and diffusion-weighted imaging does not show definite restriction in the peripheral portion **(H-I)**. **(J)** However, there was still increased cerebral blood volume on the relative cerebral blood volume map (arrowhead) on the dynamic susceptibility contrast-enhanced perfusion-weighted image.

A representative case is shown in Fig 3.

### Histopathologic analysis

**Fig 4:**
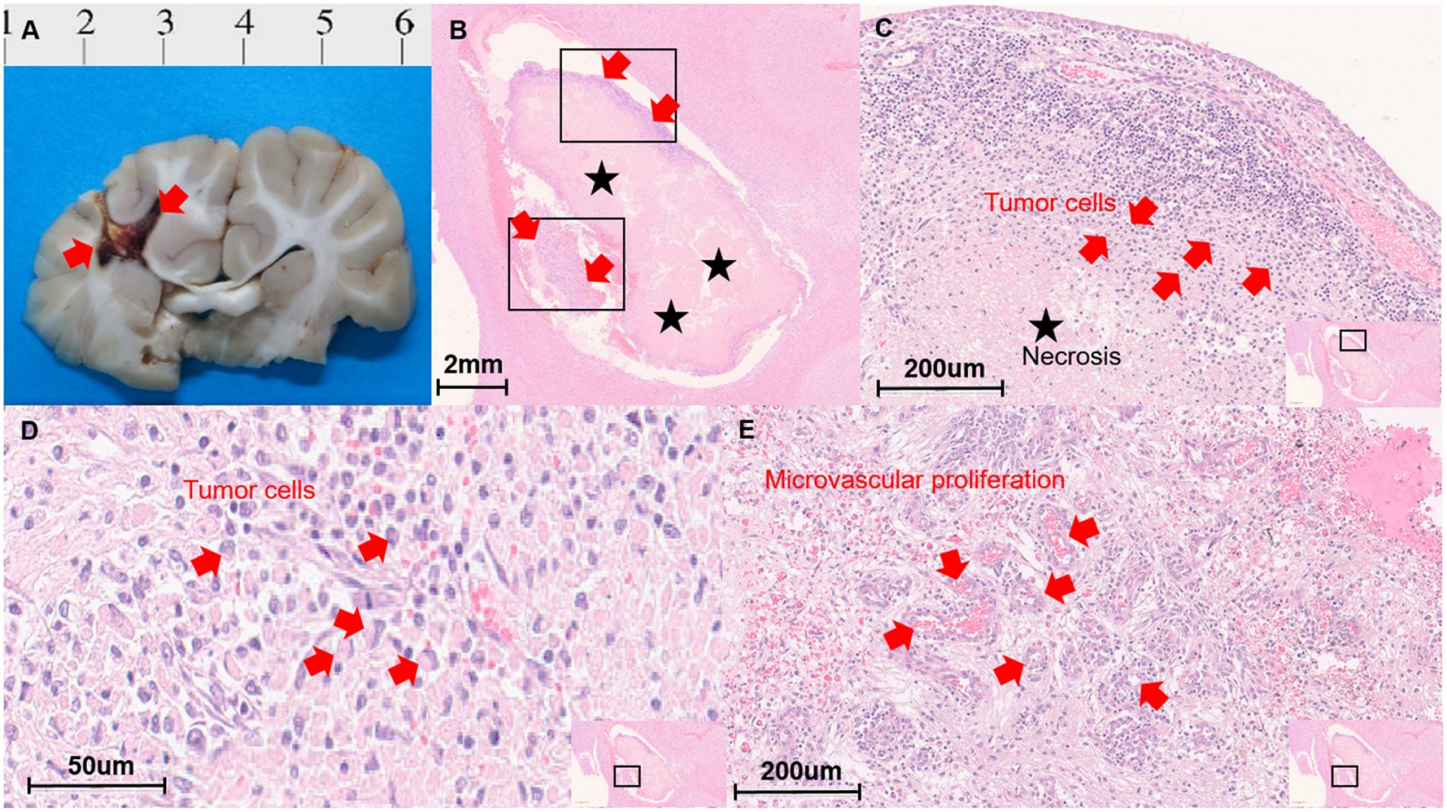
Histopathologic analysis. **(A)** The gross specimen shows a necrotic mass at the right frontoparietal white matter, the tumour implantation site. **(B)** There were scattered peripherally located tumour cells (arrows) and central necrosis (stars) on Harris’ haematoxylin solution and eosin Y (H&E)-stained images. **(C)** Multiple gemistocytic tumour cells are located between the necrotic and inflammatory portions. **(D)** On high-magnification view, the presence of glioblastoma multiform (GBM) tumour cells (arrow) was confirmed. **(E)** The characteristic microvascular proliferation in GBM tissue is also demonstrated in micrographs.

To further confirm the presence of GBM, we performed histopathological analysis (Fig 4). The number of histopathologic confirmations was 12 (92.3%) among the available specimens from 13 subjects. Two subjects’ specimens were excluded due to tissue injury during the brain extraction procedure and histologic process. The necrotic portion constituted 69.6 ± 27.5% of the tumour, and the MVD was 17.5 ± 7.5 in the viable tumour tissue. The MVD was correlated with the nCBV, which was obtained from DSC-PWI (r = 0.850, *P* = 0.004). However, the percentage of the necrotic portion did not correlate with the normalized perfusion parameters or the ADC value (all *P* > 0.05).

## Discussion

In the present study, we evaluated the imaging characteristics of the canine brain tumour model through multiparametric MRI. The human GBM-like imaging characteristics were validated through the increase in the CBV and CBF accompanied by a histopathological reference using the MVD in the canine GBM model. Therefore, it might be a possible preclinical model to assess novel diagnostic and treatment methods for human GBM.

Rodent GBM models made by intracranial or subcutaneous implantation are widely used in GBM studies and are advantageous for their high tumorigenesis and reproducible growth rate [25]. However, experimental rodent models have small brain sizes and different anatomical properties from humans, such as lissencephaly, and are thus of limited usefulness for preclinical assessment [5, 25]. The spontaneous canine GBM model can be a viable model for preclinical human cancer research. In fact, the spontaneous canine GBM model is essential for understanding the pathophysiology of tumours and developing new therapeutics in immunocompetent conditions that simulate human anatomical conditions. However, researchers cannot use spontaneous canine GBM as the experimental model due to a diagnosis time point for GBM that is too late, a very low incidence, or the emotional obligation to treat the dogs as companions [6]. Additionally, previous studies have mentioned that spontaneous tumours could be heterogeneous in nature regarding location and size [26–29]. The xenograft canine GBM model cannot overcome the limitation of using the immunocompromised rat model regarding host immunity. However, the sufficient brain size of the canine model indeed helps the development of novel imaging and treatment devices, which are critical techniques needed to diagnose and treat GBM patients. If a xenograft canine GBM model can grow to a specific size at a particular location, it can contribute to this technological advancement for GBM treatment despite the inherent immune-compromised problem.

Several attempts have been made to establish a reproducible model of human GBM in large animals. Whelan et al. [30] reported the successful development of a canine glioma model located at the posterior fossa with intracerebral injections of tumour cells and MRI scans. Krisht et al. [16] also reported a cavernous sinus tumour model in dogs with gliosarcoma cell lines harvested from nude mouse tumours. A porcine model was recently reported using U87 human GBM cell implantation [5]. The common feature among successful canine brain tumour models was that immunosuppressive agents were essential for establishing tumour cells in a large animal model. Immunosuppressive treatments that prevent cell rejection are needed to maintain exogenously injected tumour cells [31]. One such immunosuppressive therapy regimen involves a combination of cyclosporine, azathioprine, and steroids, a “triple therapy” used to prevent cell rejection in organ transplantation. Two previous studies used the following dosages for this triple therapy: cyclosporine (100 mg orally twice daily), azathioprine (50 mg orally twice daily), and prednisone (10 mg orally twice daily) [16, 30]. Therefore, we adjusted and administered immunosuppressive treatment before tumour implantation and through the end of the study at the mentioned dosages.

The MRI features of human GBM typically show a large necrotic mass with thick and irregular enhancing margins with peritumoural oedema on conventional sequences [12]. However, conventional MRI lacks pathophysiological specificity and detail and has limitations in diagnosing and evaluating the histopathological characteristics of human GBM [32]. Therefore, both DWI and DSC-PWI have been applied in the clinical imaging of human GBM as surrogate markers for tumorigenicity and angiogenesis, reflecting GBM grade and prognosis [19, 33]. Previous studies that mentioned spontaneously arising GBM in the canine model reported that the imaging and pathologic features of spontaneous GBM were similar to those characteristics of human GBM [34, 35]. However, these studies did not use the current clinical imaging MRI technique using DWI and DSC-PWI but only showed necrotic peripheral enhancing lesions on the conventional MRI technique. Microvascular proliferation with angiogenesis in the early tumorigenesis and angiogenesis of GBM requires DWI and DSC-PWI because these pathologic changes might not be demonstrated with conventional MRI techniques. In particular, DSC-PWI can show increased CBVs in tumours due to the high density of immature tumour vessels around the tumour and in disrupted blood-brain barriers [19]. Therefore, this approach could be used to characterize pathophysiology and haemodynamics during tumorigenesis in a newly developed canine GBM model. In addition, when the newly developed therapeutic device or method is applied to the canine GBM model, these MRI techniques might show the pathophysiologic changes and the therapeutic effect of those on GBM.

In our study, there was higher CBV and CBF in the canine GBM than in the normal-appearing white matter, which is similar to the imaging findings in human GBM. On serial follow-up MRI, the tumour’s peripheral enhancement, although reduced in size, remained for at least two weeks. It may be reasonable to assume the presence of a tumour given the persistent irregular enhancement and increase in CBV and CBF, which was also related to histopathological findings, despite the decreasing size of the enhancement. DSC-PWI is known to estimate the tissue MVD by measuring the CBV [36]. Our canine GBM model also demonstrated a significant correlation between the MVD and nCBV values, indicating that pathophysiological changes during tumour formation were successfully demonstrated in the canine GBM model. Additionally, the total voxel value of the tumour provided objective perfusion data within the entire tumour range, providing better quantitative accuracy.

However, in terms of the ADC values, we could not demonstrate a significant difference between the GBM and normal-appearing white matter tissue or in the nADC values on the serial follow-up images. The ADC values may reflect tumour cellularity, with higher values in cystic areas than in the solid component of the tumour [18]. The lack of a difference in the ADC values is one of our study’s limitations. In this study, ROIs were drawn along the tumour boundaries within the high-signal intensity areas on CE-T1WI. The necrosis, cystic portion, or oedema within the tumour might be a confounding factor for ADC measurement [37]. Nevertheless, the histopathologic analysis showed dysmorphic and sustained tumour cells with a large, internal necrotic portion.

Several limitations of the present study should be mentioned. First, we could not assess the appropriate blood concentration of cyclosporine in the development and maintenance of the induced tumours in the canine GBM model. Additionally, long-term data from more than two weeks postsurgery could not be obtained because of cyclosporine-induced gingival hypertrophy. Precise dose adjustment during the follow-up period is needed. However, an extended follow-up period might not be necessary, especially in the case of experiments to evaluate the potential of novel materials and device technologies for human GBM [38]. The canine GBM model, which can mimic the human brain’s size and is similar to humans, is more helpful in assessing novel technical development because it has sufficient brain size, unlike rodent models, despite the short-term follow-up. Second, sophisticated surgical stereotactic devices are required to determine the exact tumour insertion site and increase the surgery success rate. There were two subjects in which leptomeningeal seeding resulted from intraventricular extension due to the deep location of the tumour implantation site on the follow-up MRI. Accurate positioning through imaging-guided tumour implantation might improve the success rate of tumour implantation. Third, the T2-weighted images from the DSC-PWI technique suffered from susceptibility artefacts [39]. There was a relatively high rate of inappropriate DSC-PWI data because of the surgical material used for the cortex. When choosing surgical material to prevent bleeding at the surgical site, it would be better to select a material that would lead to fewer susceptibility artefacts to improve the image quality. Fourth, the image acquisition of DSC perfusion did not follow the current consensus protocol for brain tumour imaging regarding FA and contrast agent preload [40]. The image acquisition protocols with an FA of 90° and no contrast agent preload might maximize the confounding T1 effects of contrast agent leakage. Additionally, we used the gamma-variate function and correction of contrast agent leakage through options in the NordicICE software package. Fitting to a gamma-variate function might force the postbolus signal to baseline, making any subsequent leakage correction less meaningful in brain tumour image data. We believe that the recommended imaging protocol and analysis method could improve the image quality of DSC perfusion MRI. However, in this study, the algorithm and software in the perfusion parameter calculation were consistently applied to compare the difference in perfusion status between the tumour and normal-appearing white matter. Finally, the results of decreasing tumour size and perfusion parameters over time might not properly reflect a treatment effect observed clinically. Although we assessed the presence of tumour cells in the obtained tissue and obtained 92.3% tumour presence among the available specimens from 13 subjects, there might be a discrepancy between the microscopic and macroscopic xenograft GBM in a large animal model. Although faster than in humans, the progression of cancer in dogs is slower than in most murine models, so it may take some time for large animal trials to provide informative data [6, 41]. Indeed, there are still limitations, such as the need to take immunosuppressants to maintain a macroscopic tumour mass. However, our data showed no significant difference between the 1-week and 2-week follow-ups regarding nCBV and nTTP values. A xenograft canine model might be possible to use to evaluate the efficacy of a new treatment method for one or two weeks.

In conclusion, this study showed that the canine GBM model exhibited perfusion imaging characteristics similar to those of humans and histopathological features to human GBM. The canine GBM model can help clinicians understand the pathophysiology of GBM, and we believe that it can be used as a potential experimental model for diagnostic and therapeutic assessment for GBM.
